# Mummification in a forensic context: an observational study of taphonomic changes and the post-mortem interval in an indoor setting

**DOI:** 10.1007/s00414-023-02986-3

**Published:** 2023-03-21

**Authors:** Ann-Sofie Ceciliason, Björn Käll, Håkan Sandler

**Affiliations:** 1 Forensic Medicine, Department of Surgical Sciences, Uppsala University Hospital, Uppsala University, SE-751 85 Uppsala, Sweden; 2 Department of Forensic Medicine, The National Board of Forensic Medicine, Box 1024, SE-751 40 Uppsala, Sweden

**Keywords:** Forensic taphonomy, Desiccation, Mummification, Human remains, Post-mortem interval estimation

## Abstract

The objective of this study was to evaluate the presence of mummification in an indoor setting, with an emphasis on the forensic perspective. A dataset of 102 forensic autopsy cases was assessed for distribution of desiccation of skin and soft tissue (i.e., subcutaneous fat and musculature) and for moist decompositional (i.e., putrefactive) changes. Further, possible correlation with the post-mortem interval (PMI) was evaluated, as well as the effects of clothing coverage of the body. The results indicated that yellow to orange parchment-like desiccated skin was found at significantly shorter PMIs than reddish brown to black leathery desiccated skin, even when soft tissue desiccation was included in the comparative analysis. Clothing appeared to have a significant decelerating effect on the extent of desiccation on the legs, but findings in regard to whole body or torso/arms were inconclusive. A large variation in PMIs was evident as regards fully desiccated skin (PMI 18–217 days), indicating difficulties in PMI estimation due to a variable repressive effect on the decompositional process per se in an indoor setting. For the specific case in forensic practice, no definite conclusion can be drawn from the observed desiccation changes to the PMI. One way forward might be creating a systematic and standardized method for describing different desiccation types, as well as other cooccurring decompositional changes and how they relate to the PMI, as a foundation for a future quantification model.

**Supplementary information:**

The online version contains supplementary material available at 10.1007/s00414-023-02986-3.

## Introduction

Desiccation is a rather sparsely studied process, especially in a forensic context, with regard to estimation of the post-mortem interval (PMI). Most published studies are case reports describing desiccation of human remains in an indoor setting [1–7]. Leccia et al. [8] presented a retrospective study of 35 mummified cases where the degree of desiccation in each case was estimated, using the “rule of nines” for burns. The cases were from both indoor and outdoor settings and the PMI of the cases varied considerably, from a few weeks to several years. Connor et al. [9] presented a total body desiccation score method based on 40 donated human remains, placed in an outdoor setting and observed over a long time period. This novel scoring model might improve PMI estimations in cases with desiccation. Still, it is not fully known how the desiccation process differs between an indoor setting and an outdoor setting. Findings of mummified human remains in an indoor setting are not rare [8] and are often seen as a sign of social isolation [6].

Though decomposition is considered to be a continuous process, it is commonly divided into different stages [10]. These can include fresh, early decomposition, advanced decomposition, skeletonization, and decomposition of the bones [11]. A dead body may be in different stages in different anatomical regions – desiccation may occur at the same time as moist decomposition and partial skeletonization [12, 13].

The terms mummification and desiccation are sometimes used interchangeably, without any clear definitions. This may complicate the interpretation of human remains and PMI estimation. The cell enzymes and microorganisms present in a dead body require water to function, and if the supply of water is reduced, the rate of decomposition will decline [13, 14]. Desiccation, i.e., loss of tissue moisture, is therefore an important process to study. Mummification is the point at which the decompositional process ceases [15]. Such preservation of soft tissue can occur naturally, as seen in the Tyrolean iceman “Otzi” [16, 17], bog bodies found in swamp lands [18], or the “modern mummies” of today [8, 19], or through human manipulation, as in the case of Egyptian mummies [20].

Extrinsic environmental factors, such as aridity, circulation of air, and climate, are commonly seen as having a strong impact on soft tissue desiccation [10, 19, 21–23]. An enclosed and ventilated space could favor desiccation [24]. Another effect of enclosed places is the partial or complete preclusion of insects [25]. Studies have indicated that restricted or non-existing insect activity may result in optimal conditions for desiccation [19, 23].

Clothing has been indicated to have an association with development of desiccation [1, 20]. Aufderheide [21] has argued that if skin is pressed tightly against another part of the body or against tight clothes, this can prevent water evaporation. It is suggested that desiccation is more likely to occur in smaller bodies, with a higher ratio of skin to underlying tissue and less body fat [24, 26], though there are conflicting views on this [22]. Prominent body parts or parts with more skin in relation to the amount of underlying tissue, e.g., fingers and outer ears, are more prone to desiccation [19, 20]. Emaciation is also thought to favor desiccation [1]. The development of mummification is considered to take weeks, if not months, and once mummification is reached, the body can be preserved in that state for an extended period of time [10, 19, 24]. Soft tissue preservation may interfere with the estimation of PMI and an experimental study has shown further development of desiccation and tissue changes after a PMI of 9 years, suggesting that this is an ongoing process [27]. More knowledge concerning the sequence and rate of desiccation of human remains in an indoor setting is needed to enhance the accuracy and precision of PMI estimations.

The overall objective of this study was to investigate *and describe* human remains, with a presence of desiccation, found in an indoor setting. The ambition was to evaluate the possibility to create a useful tool for forensic pathologists/anthropologists in assessing desiccated (or mummified) human remains. The emphasis was on the body’s exterior, e.g., extent of desiccated skin, type, and patterns of desiccation, with a *future* scoring-based model in mind for quantification purposes.

## Materials and methods

### Study sample

This study compiled 102 forensic autopsy cases collected non-consecutively from 2010 to 2019 at the Departments of Forensic Medicine in Uppsala and Gothenburg, Sweden. The inclusion criteria were: human remains found indoors in an apartment or house, of adult age (> 18 years), with some presence of desiccation. Further, a PMI had to be possible to estimate based on information other than the decompositional changes, such as witness statements or findings on the scene of death (e.g., prescriptions, oldest mail/newspaper, phone call, etc.) included in a police report. Cases with only desiccated fingers, toes, nose tip, lips, or external parts of the ear were not included as such changes sometimes appear in cases with no other moist decomposition or desiccation changes visible on the body. It would therefore be difficult to determine if these changes were artefactual and if they had arisen before or during storage in the morgue.

### Collection of data

The data in this study were originally collected during routine forensic investigations. They included last time known to be alive (used to estimate PMI), days in storage at the morgue facility, gender, age at death, and presence of clothes/coverings. Estimation of PMI: Circumstances described in police reports, such as statements from witnesses concerning last seen alive or other evidence (e.g., prescriptions, phone calls), were used to estimate PMI in each case. This may have result in overestimation of the true PMIs. Morgue time: The interval during which the dead body was stored at a morgue was calculated from date of discovery to date of autopsy. Clothes/coverings: Presence of textiles was collected from the police report and/or at the autopsy. Each case was anonymized and given a specific case ID.

#### The indoor setting

In the forensic routine investigation, measurements regarding the temperature and humidity at the death scene was not commonly recorded. This was done exclusively in cases of suspected homicide. Our data set only consisted of routine forensic cases. A limitation is that we lacked information about the exact temperature and humidity for each case in the dataset. However, in Sweden, indoor environments are well-regulated. The indoor temperature recommended by the Swedish Public Health Authority is + 20–23 °C [28], and the Swedish construction standards require an indoor temperature within the range + 16–26 °C [29]. The humidity indoors in Sweden is commonly within the range 40–60%. During the summer period, the humidity increases to above 40% and then falls sharply in the autumn [30]. In Swedish homes, humidity can drop below 10% during the winter [30].

### Assessing decompositional changes

**Fig. 1:**
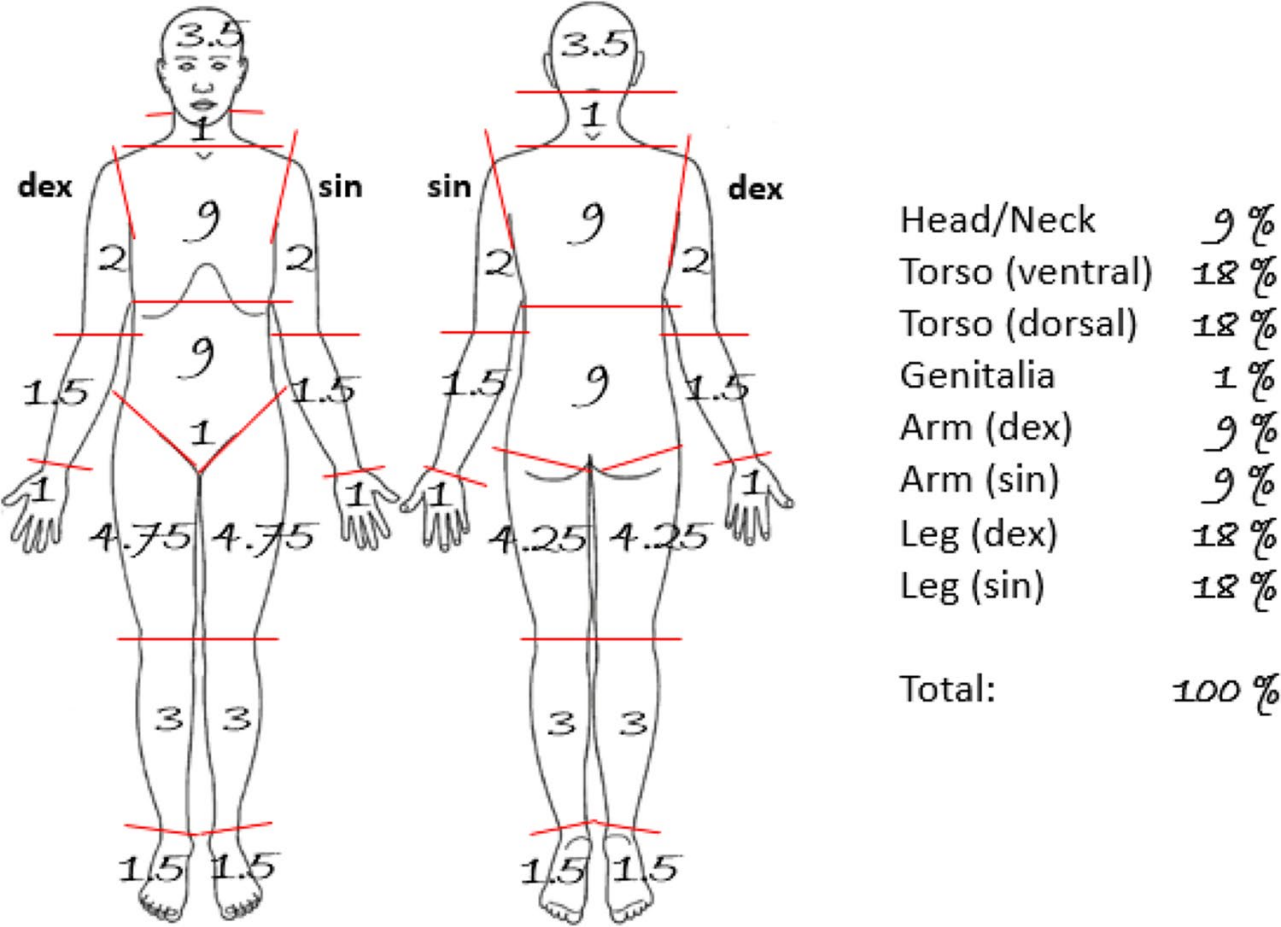
Body template used for calculation of body surface affected by desiccation or moist decomposition. Modified after Lund and Browder [31] for assessment of desiccated skin. The anatomical regions (32 in total) were assessed separately and then added together

The presence of decompositional changes was assessed *by visual inspection* during the forensic autopsy and were noted on a body template displaying both front and back views of the body. The following decompositional changes were recorded: skin slippage, blisters, discoloration of skin, marbling of skin, bloating, desiccation, soft tissue loss, and bone exposure. Also noted were some changes indicating the level of moistness/dryness inside the body, such as purging of decompositional fluid from nose/mouth, fluid in body cavities, and absence of blood in heart and/or femoral vein. Presence of insect activity or mold on the body surface was noted on the body template. The percentages of body surface affected by desiccation, moist decomposition (putrefaction with liquefaction of soft tissue), and unaffected were estimated and noted on the body template. This was done using a modified version of Lund and Browder’s burn injury degree chart [31]. In the modified chart, the trunk makes up 37%, the arms 18%, the legs 36%, and the head and neck 9% (see Fig. 1 for details).

### Classification of desiccation changes

In a previously published study, two main types of desiccation characteristics were suggested to be present in human remains found in an indoor setting, as well as a mixture of the two [32]. These are parchment-like desiccated skin, commonly yellow to orange in color, and leather-like desiccated skin, often a reddish brown color. Based on the above, the desiccation changes likely to be observed in our study were classified into three types: type 1 – parchment-like desiccated skin, type 2 – leathery desiccated skin, and type 3 – additional desiccation of underlying soft tissue, such as musculature and fat tissue (hard or stiff in texture). Their presence was recorded on the body template and discoloration of the skin was also noted. We assumed it could be possible to observe all three types on the same body.

### Processing of data

Data noted on the body templates were compiled in an Excel file (Microsoft Excel version 16.42). Insect activity and presence of mold was dichotomized (present/not present). No further categorization of site and amount of insect or mold activity was made. This limitation of the study was due to lack of access to the death scene for further entomological analysis as well as a small sample of cases within the data set with presence of insects or mold.

For the descriptive part of the study, variables related to desiccation of the skin and soft tissue were dichotomized as present or not present. In addition, the observed desiccation changes were subcategorized (see Fig. S1) to study their occurrence within the sample. Three nonexclusive categories were used, meaning that other processes could appear simultaneously, but were not specified further (leathery, parchment-like, and soft tissue desiccation). Three exclusive categories were also used, where only one process was present (exclusively leathery, parchment-like, and soft tissue desiccation), as were four categories with specified combinations (leathery in combination with parchment-like, leathery in combination with soft tissue desiccation, parchment-like in combination with soft tissue desiccation, and all three types). A small number of cases (n = 4) exhibited desiccated skin that was registered as leathery to parchment-like, meaning that there were difficulties in classification. Cases with this type of desiccation were included in both categories (leathery and parchment-like). Since a body could display more than one type of desiccation, comparisons between groups were difficult. To resolve this, the cases that exhibited only leathery or parchment-like desiccated skin were compared. However, the number of cases presenting only one type of desiccation was small in our dataset. Hence, to expand the comparative analyses, cases with a presence of soft tissue desiccation were also included. This resulted in the following possible comparative analyses of desiccation types: exclusively leathery desiccation versus exclusively parchment-like desiccation AND exclusively leathery desiccated skin with or without soft tissue desiccation (leathery ± soft tissue desiccation) versus exclusively parchment-like desiccated skin with or without soft tissue desiccation (parchment-like ± soft tissue desiccation). No cases with exclusively soft tissue desiccation (i.e., without desiccated skin) were found in the dataset. The cases were originally prospectively assessed during autopsy (by the authors: ASC, HS), but for this specific study, all the cases were retrospectively reassessed (i.e., the documentation from the autopsy including body charts and photographs) and the percentage of desiccated skin was calculated (by the authors: ASC, BK). No documentation of the water content of the skin or tissue was performed during the forensic investigations. Therefore, we do not know if the end stage (mummification) was reached in any of the cases with 100% desiccated skin surface.

To evaluate the effect of covering textiles on the development of desiccation, Guerra’s clothing score model [33] was used. This gives a score to each covering material depending on its estimated insulating capability. For each case in the dataset, the scores for the torso/arms, legs, and whole body were calculated. In five cases, textiles appearing in the sample were not included in Guerra’s scoring model and the model therefore had to be slightly modified to fit our specific circumstances (see Table S1 for details).

The cases were also categorized based on whether they had any textile coverage (including blankets etc.) or no textile coverage (naked) of the trunk and the legs, respectively. Any textile coverage of the trunk was then subdivided further into one textile on the upper body but no jacket, multiple textiles on the upper body but no jacket, and jacket (not specified further). This categorization was necessary to be able to evaluate if coverage of textiles had any effect on the desiccation process, and if there was a difference in desiccation between single and multiple layers of textiles. As regards coverage of the legs (feet not included), one analysis was made when comparing naked/only underwear with partial coverage of legs, one analysis when comparing naked/only underwear with complete coverage of legs, and one analysis when comparing naked/only underwear with both partial and complete coverage of legs. One case was excluded from the descriptive statistics and another case was excluded from the inferential analysis due to incomplete data.

### Statistical analysis

The statistical analysis was carried out using IBM SPSS Statistical Subscription Build 1.0.0.1508. Normality assessment of data was performed by inspecting histograms and QQ-plots, as well as using Shapiro–Wilk’s test (p-value < 0.05 was indicated as a non-normal distribution). Since none of the correlation analyses in this study met parametric assumptions, Spearman’s rank correlation was used. In the bivariate analyses of this study, Mann–Whitney U tests were performed, since parametric assumptions were not met. A population pyramid was then used to assess similarities in distribution between the categories, if there were significant differences between them. Lastly, Kruskal–Wallis tests were performed for studying the extent of the upper body surface affected by desiccation depending on how many textile layers were present. The significance level was set at a p-value of 0.05 for all statistical tests.

## Results

### Forensic autopsy cases characteristics

A total of 102 cases were assessed, where the PMI ranged from 3 to 217 days (median 21 days). The median storage time at the morgue facility was 4 days (range 0.5–18 days). The distribution female: male was 1:4 and median age at death was 65 years (range 21–91 years). The percentage of desiccated skin, i.e., the extent of the body exterior exhibiting desiccation, ranged from 3.75 to 100%.

### General description of decompositional changes

**Fig. 2:**
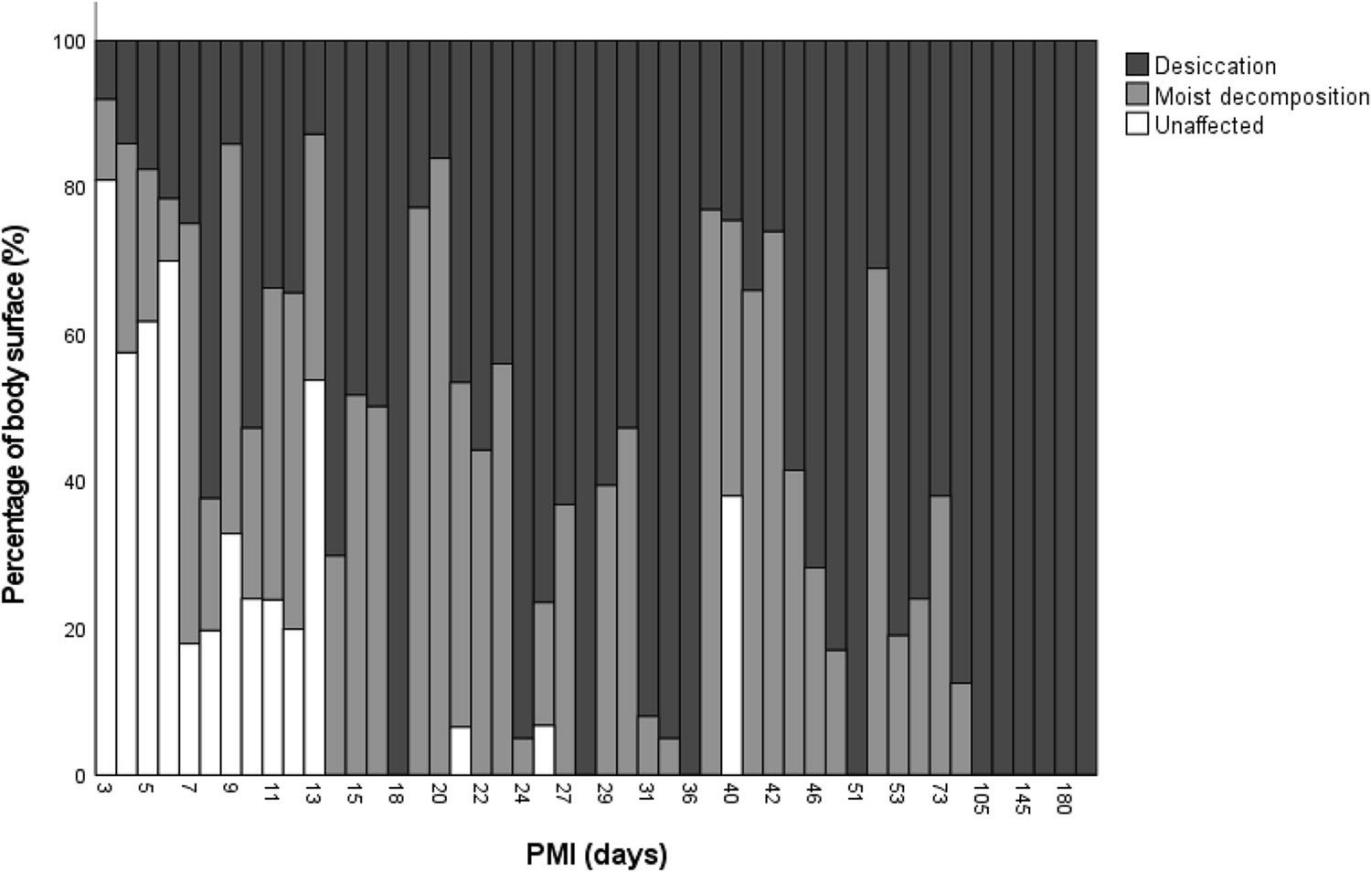
Histogram of percentual estimation of body surface in the 102 forensic autopsy cases; a) affected by desiccation, b) affected by moist decomposition, and c) unaffected, grouped by post-mortem interval (PMI). Each of the 48 bars represents one unique PMI value. If multiple cases had the same PMI value, the mean percentual estimation of affected body surface for that PMI was calculated. The x-axis is shown as a categorical scale

The cases displayed decomposition to a varying degree, as well as unaffected areas. Moist decompositional changes include skin slippage, skin blisters, discoloration and/or marbling of skin, disintegration of skin/soft tissue, soft tissue loss, and bone exposure. Of the 102 cases with desiccation, 81 cases also displayed moist decomposition. All these 81 cases exhibited moist decompositional changes within the trunk, with the legs affected in 61/81 cases, the head affected in 60/81 cases, and the arms affected in 54/81 cases. The distribution of desiccation and moist decompositional changes as well as unaffected areas are shown in Fig. 2. Desiccated skin was seen as early as at PMI 3 days and 100% coverage of the body surface was reached at PMI 18 days. However, there were large variations in the distribution of desiccation and moist decomposition. Unaffected areas were commonly seen at short PMIs, but there were a few cases with unaffected areas of body surface at longer PMIs (21, 25, and 40 days). Soft tissue loss (disintegration of the skin and underlying fat and musculature) and bone exposure was seen in 26 and 6 cases, respectively, mainly affecting the head (face), neck, and upper torso. These cases commonly displayed insect activity.

### Observed desiccation changes

**Fig. 3:**
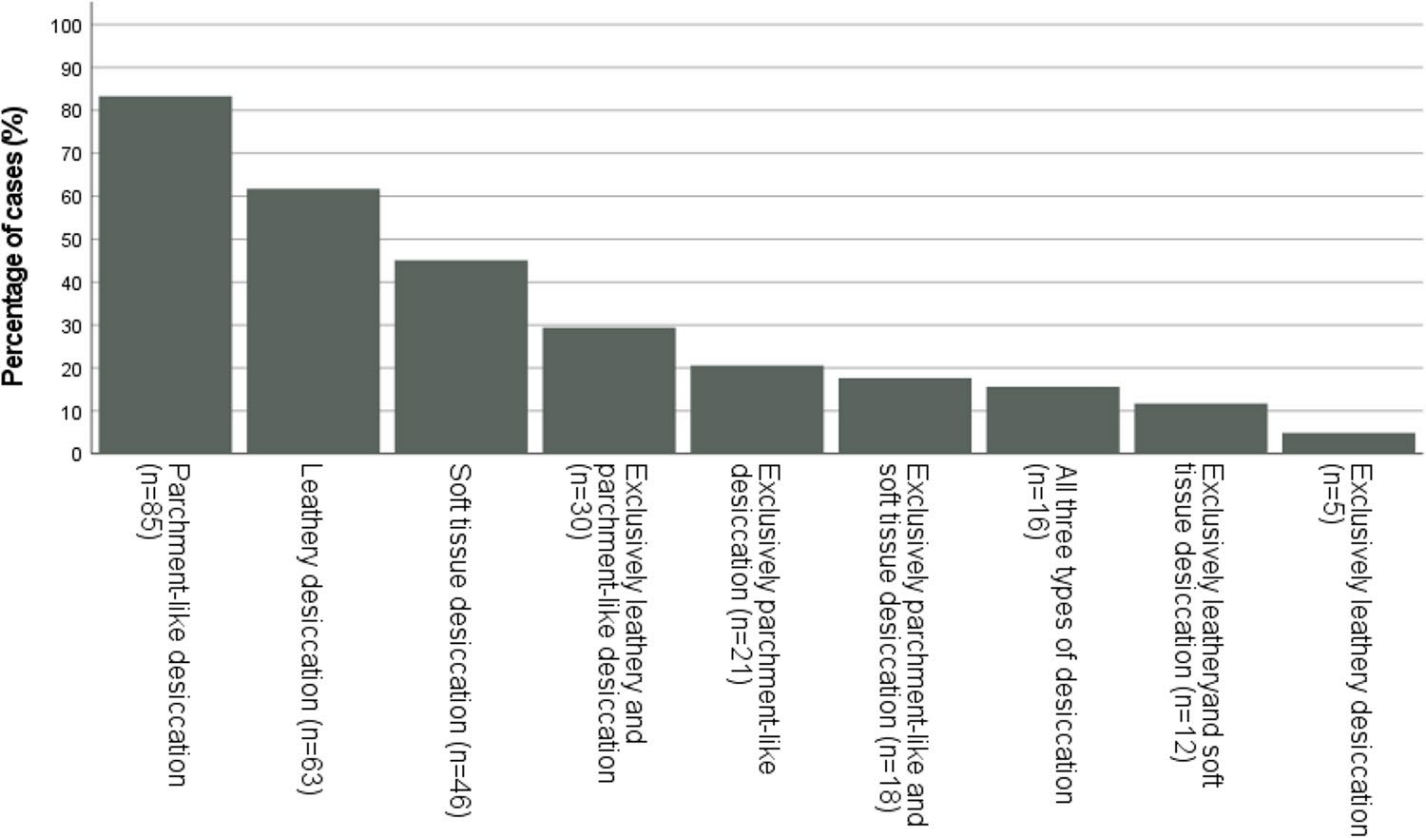
Bar chart showing percentage of cases exhibiting each type of desiccation observed within the dataset. Cases with “leathery desiccation” could host another desiccation process at the same time, but cases with “Exclusively leathery and parchment-like desiccation” did not exhibit any soft tissue desiccation. No cases with only soft tissue desiccation were observed in the dataset

The distribution of desiccation varied considerably within the dataset, as shown in Fig. 2. The distribution of different desiccation changes also varied (as seen in Fig. 3). The most frequently observed type was parchment-like desiccated skin, followed by leathery desiccated skin. A limited number of cases also displayed soft tissue desiccation (i.e., desiccated subcutaneous fat and musculature). In several cases, a combination was seen.

### Leathery desiccated skin

Leathery desiccated skin was observed in 63 of 102 cases. In five of these cases, leathery desiccated skin was the only desiccation change observed. Leathery desiccated skin felt thicker than parchment-like desiccated skin. The discoloration of the skin varied, encompassing different combinations of black, red, green, orange, violet, blue, and brown. Most frequently seen was a combination of red and brown or black and brown.

### Parchment-like desiccated skin

In 85 of 102 cases, parchment-like desiccated skin was observed. In 21 cases, parchment-like desiccated skin was the only desiccation change observed. The texture was like that of thin skin, often pellucid with a prominent network of fine visible blood vessels and small wrinkles.

The most commonly observed discolorations were yellow to orange. However, a range of nuances was seen, including brown, black, green, red, grey, violet, and cerise.

In 4 of the 102 cases, the skin displayed a mixture of leathery to parchment-like desiccation and could not be distinctly classified as either parchment-like or leathery, or had patches of both types. This was most commonly observed on the abdomen and thighs.

### Soft tissue desiccation

**Fig. 4:**
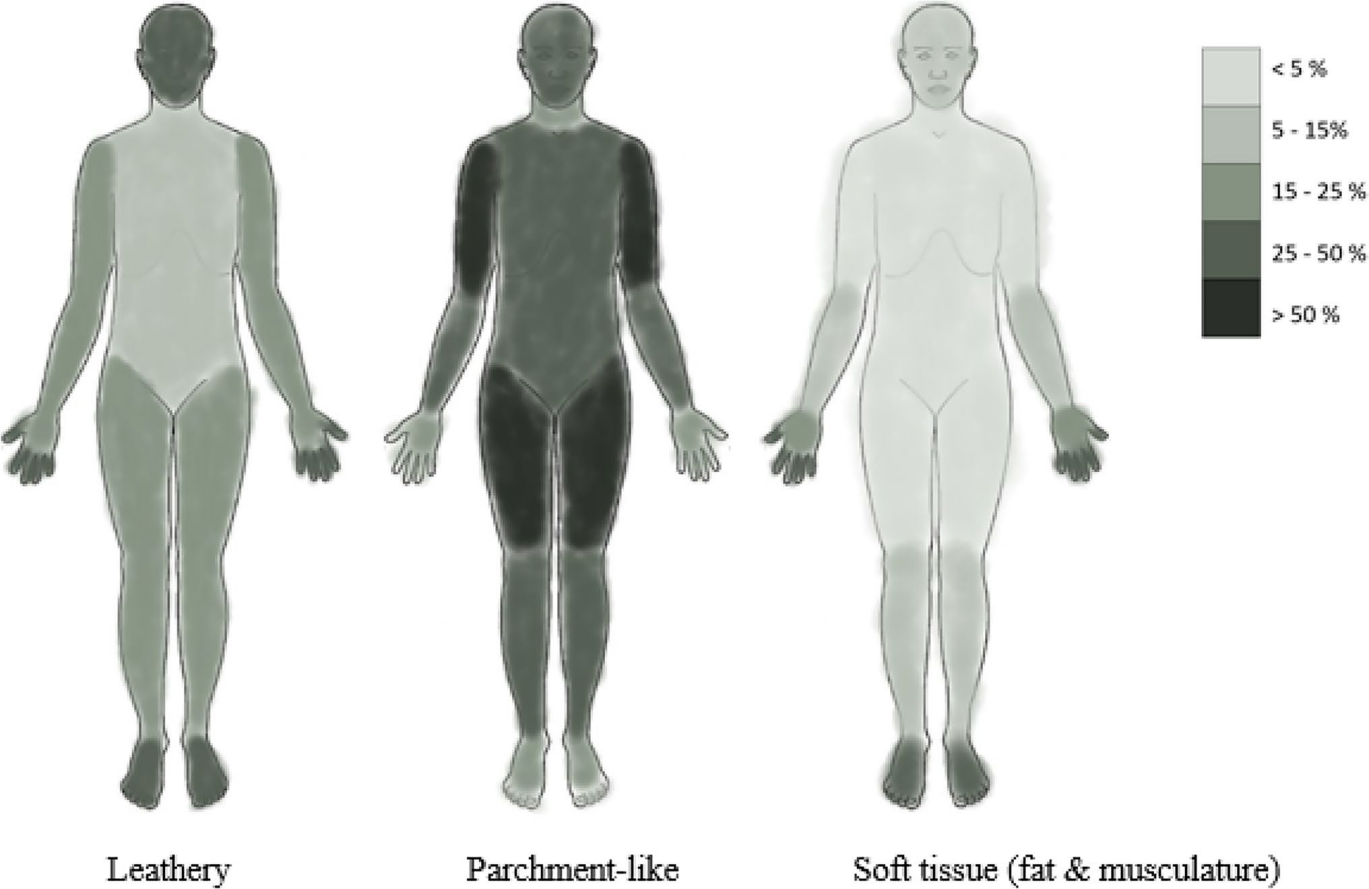
Distribution of the different desiccation changes expressed as percentages of total number of cases. Ventral and dorsal side combined and represented in one frontal body chart. Details can be found in Fig. S2 to S4

Desiccation of the underlying tissue (i.e., subcutaneous fat and musculature) was also investigated. However, we did not assess possible desiccation changes of the internal organs. Of the 102 cases, 46 cases displayed areas of soft tissue desiccation. The limbs were commonly affected, especially the hands and feet (as seen in Fig. 4).

When evaluating anatomical regions for the different desiccation types, a pattern could be distinguished. Figure 4 shows a schematic representation of leathery, parchment-like, and soft tissue desiccation. The distribution of leathery desiccated skin seemed similar to that of soft tissue desiccation, mainly affecting the limbs and the head. Parchment-like desiccated skin was often found on upper arms and thighs.

When comparing the cases divided into 3 ranges based on desiccated area of body surface (≤ 40%, 40–80%, or > 80%, as presented in Table 1), age, and morgue time did not differ substantially between these ranges. PMI increased with higher percentual area of desiccated skin. Signs of insect activity as well as signs of molds were found in a few cases, regardless of the extent of desiccation. Moist decomposition features (e.g., decompositional fluid in body cavities) were seen even in cases with > 80% of skin surface desiccated. On the other hand, cases with minor desiccation changes (≤ 40%) could lack decompositional fluids in nose/mouth (purge) and body cavities and be without blood in the heart and femoral vein.

**Table 1:** Comparison of three groups based on the total area of body surface affected by desiccation (≤ 40%; 40–80%; > 80%). Normally distributed variables described as [mean ± SD], not normally distributed variables described as [median (IQR; range)], and dichotomous variables expressed as a quotient

Area of body surface desiccated (%)	≤ 40 (n = 47)	40–80 (n = 23)	> 80 (n = 32)
Female/male	11/47	6/23	6/32
Age at death (years)	61.8 ± 14.4	62.7 ± 11.5	63.6 ± 10.8
PMI (days)	13.0 (14.0; 49.0)	23.0 (18.0; 65.0)	30.5 (43.0; 209.0)
Morgue time (days)	4.0 (3.0; 17.5)	5.0 (1.0; 9.5)	4.0 (3.0; 11.0)
Guerra’s clothing score	4.3 ± 2.9	3.2 ± 3.1	2.7 ± 2.6
Naked on upper body	9/47	9/23	12/32
Naked/only underwear on legs	17/47	14/23	18/32
Fully naked/only underwear	5/47	6/23	11/32
Cases with signs of insect activity	13/47	5/23	8/32
Cases with signs of mold activity	6/47	4/23	4/32
Cases with decompositional fluid in nose or mouth (purge)	22/47	9/23	10/32
Cases with decompositional fluid in body cavities	21/47	9/23	10/32
Cases without blood in heart and/or femoral vein	18/47	12/23	19/32

As Table 1 shows, the cases seemed to be rather heterogenic in nature, with a broad range of post-mortem changes. This remained apparent when looking only at the cases that had 100% desiccated skin (n = 21). Even when the skin was desiccated and some soft tissue desiccation was present, we observed putrefactive changes, such as presence of decompositional fluids in body cavities, bloating, and greenish discoloration of the skin. The PMI in this group of cases was 18–217 days (median 31 days).

### Desiccation and correlation with PMI

**Fig. 5:**
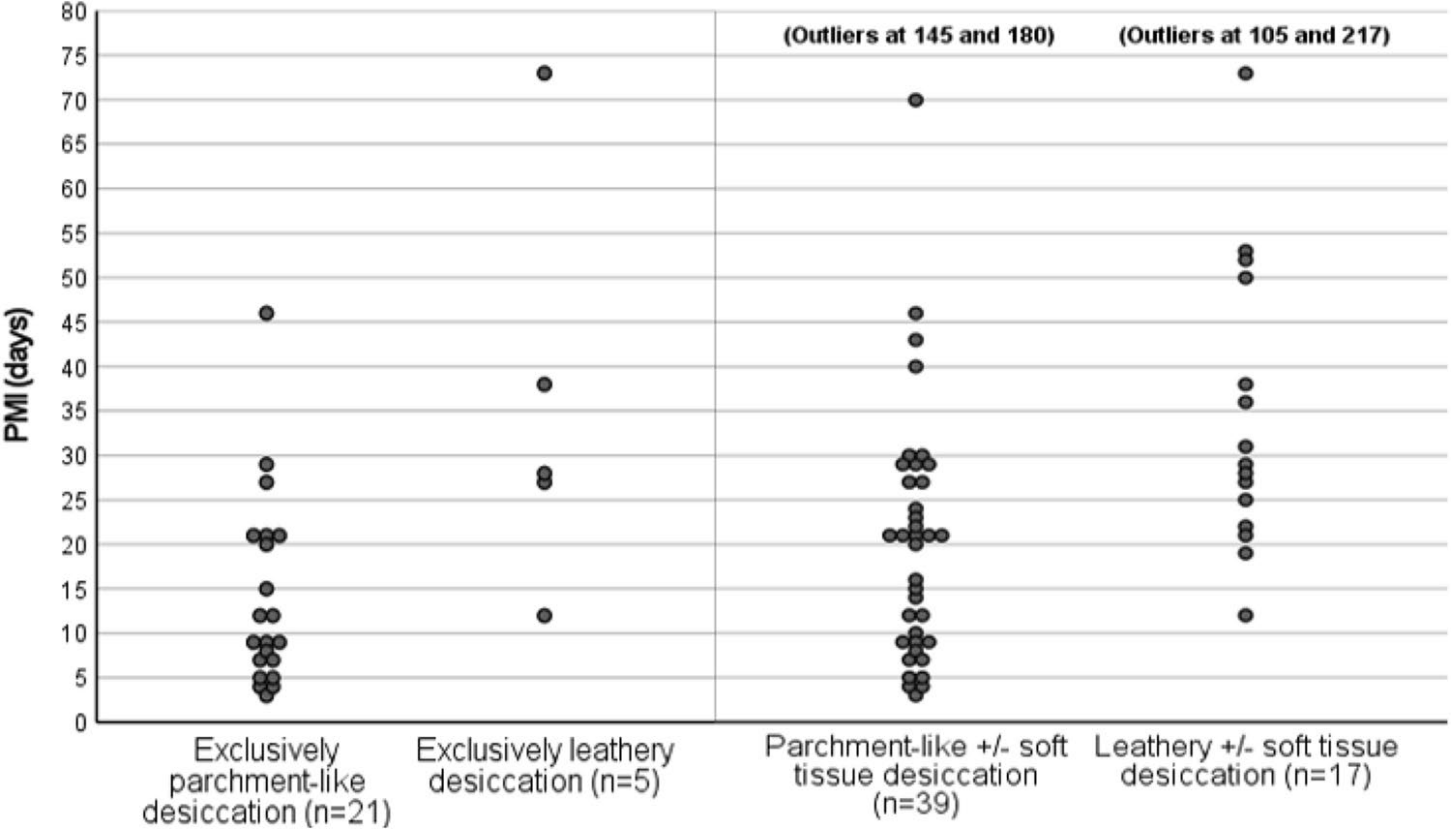
Scatter plot showing distribution of post-mortem interval (PMI) relative to the type of desiccation the cases exhibited, e.g., “Leathery ± soft tissue desiccation” shows the cases that exhibited leathery desiccation on the body surface, with or without underlying soft tissue desiccation

Spearman’s correlation reached a coefficient of 0.560 (*P* < 0.001) between the extent of body surface affected by desiccation and the PMI, indicative of a moderate significant relationship. As expected, the percentage of desiccated skin increased with longer PMIs. As shown in Fig. 5, there were some outliers. Examples include a case with PMI of 8 days and 98% coverage of desiccated skin and cases exhibiting only 8.25% desiccated skin at PMI 30 days or 31% at PMI 52 days. No linear relationship between PMI and percentage of body surface desiccated could be found in the dataset.

### Correlation between PMI and type of desiccation

All three types of desiccation observed were present in both cases with short PMIs and cases with extended PMIs. Leathery desiccated skin was observed at PMI 6 days, at the earliest, parchment-like desiccated skin at PMI 3 days, and soft tissue desiccation at PMI 8 days. When comparing the PMI between cases exhibiting exclusively leathery desiccated skin (mean rank = 21.10; *n* = 5) and cases exhibiting exclusively parchment-like desiccated skin (mean rank = 11.69; *n* = 21), a significant difference was found (*U* = 14.5; *P* = 0.01). Cases with leathery ± soft tissue desiccation (mean rank = 38.68; *n* = 17) showed a significantly higher PMI (*U* = 158.50; *P* = 0.002) than cases with parchment-like ± soft tissue desiccation (mean rank = 24.06; *n* = 39). The distributions of PMIs within each of aforementioned groups are presented in Fig. 5.

### Effects of textiles covering body surface on desiccation

Thirteen of the 101 cases in the dataset were fully naked. Thirty cases were naked on the upper body. Among the cases with clothes on the upper body, 29 had multiple layers. Twenty-one of the cases had naked legs and 27 cases wore only some type of underwear, resulting in 48 cases classified as having legs naked. No significant correlation was found between the extent of desiccated skin and Guerra’s clothing score [33] regarding either the whole body (*rs* = -0.150; *P* = 0.14), the legs (*rs* = -0.186; *P* = 0.06), or torso/arms (*rs* = -0.177; *P* = 0.08), respectively (see further Fig. S1 and Table S2).

When comparing the total area of the trunk affected by desiccation depending on if any textile coverage was present on the upper body (n = 70) or not (n = 30), no significant difference was found (*U* = 853; *P* = 0.14). Furthermore, when comparing the extent of the trunk area affected by desiccation depending on which type of upper body clothing was present, the Kruskal–Wallis test showed no significant differences, *P* = 0.23.

Moreover, no significant difference in total area of legs affected by desiccation was observed when comparing partially covered legs (n = 9) with naked/only underwear (n = 48), (*U* = 142; *P* = 0.10), or when comparing completely covered legs (n = 43) with naked/only underwear (*U* = 813; *P* = 0.08). However, when partial and complete covering of the legs were grouped together (*n* = *52*), a significant difference was seen when compared with naked/only underwear (*U* = 955; *P* = 0.039). In that analysis, the group that had no coverage (naked/only underwear) had a median value of 68.1% of the legs being affected by desiccation, while the group with coverage had a median desiccation area of 34.7%.

## Discussion

This study presents some novel findings regarding desiccation in an indoor setting. A dataset of 102 forensic autopsy cases displaying desiccation to various degrees and in differing patterns was evaluated. The results highlighted that desiccation changes could appear at widely different PMIs, making estimation of PMI based on visual assessment of a desiccated body surface difficult. For the individual case no secure conclusion about the PMI could be made exclusively using the desiccation changes observed. The results also indicated a large inherent complexity in observed post-mortem changes and a need to better classify and describe desiccation to improve the quality of forensic investigations and possible also PMI estimations.

### When is a dead body mummified?

The word “mummified” is used for a range of different desiccation changes. However, little has been done to analyze and describe mummification from a forensic perspective. Presence of mummification interferes with PMI estimation, as well as with assessment of possible injuries and pathological changes determining the cause and sometimes also the manner of death. In this study, we have elected to use the word “desiccation” to describe the entire process, with mummification as its end stage (when the decomposition process has ceased). In a study by Megyesi et al. [34], the dead body is considered to pass through several stages of decomposition – early decompositional changes, bloating, post-bloat, moist decomposition, and mummification – before the skeletonizing stage; this means there is an accumulating decomposition, with mummification coming after moist decomposition. In our dataset, it was apparent that desiccation and moist decomposition could occur simultaneously in the same body, indicating competing processes: decomposition versus desiccation. Neither autolysis nor putrefaction leads directly to tissue dehydration [35].

### Observed desiccation changes

In this work, we classified observed changes into three different types: 1) leathery desiccated skin, 2) parchment-like desiccated skin, and 3) soft tissue desiccation, i.e., desiccation of subcutaneous fat and underlying musculature. This classification was based on the texture of the skin and its visual appearance. An observable difference was that leathery desiccated skin was more common in distal parts of the limbs and parchment-like skin was seen in anatomical regions with more subcutaneous fat, such as thighs and upper arms. Soft tissue desiccation was often seen distally, with a similar distribution pattern as leathery desiccated skin. We could hypothesize that the composition of a specific anatomical region affects the development of desiccation. The water content may vary between different body parts.

Since very few studies has been carried out analyzing the desiccation process per se, it is difficult to make comparisons with our findings. A relevant study describing desiccation in a forensic perspective is Connor et al. [9]. However, that study analyzed only outdoor cases and is therefore not fully comparable. The main difference between indoor and outdoor decomposition is that the enclosed indoor environment may limit insect accessibility and thus alter loss of soft tissue (which is mainly driven by insect activity). Most indoor cases do not reach a skeletonized stage. In the article by Connor et al. [9], the classification of the skin desiccation was as follows: drying of edges, rawhide, crenulated, pebbled mosaic*,* ending as parchment. At this end stage, the dead body is skeletonized and remnants of the skin are parchment-like. In our dataset, we did not observe any changes similar to crenulated, pebbled mosaic, or parchment. The desiccated skin that we labeled as parchment-like showed presence of soft tissues, in various states of decomposition or desiccation. This difference could be due to lack of soft tissue destruction from maggots feeding and/or exposure to an outdoor environment, if we assume that parchment(-like) means the same thing in both studies. The desiccation category rawhide [9] may be comparable with our “leathery desiccation of the skin,” but a comparison based only on photographs is not optimal. Interesting to notice is that Connor et al. [9] also included moisture categories: purge, surrounding soil wet, sweating/glistening, and desiccated. In our work, we selected the following signs of moisture content: presence of decompositional fluids from nose/mouth (purge) or in body cavities and blood in heart and/or femoral vein (absence of blood could be a sign of the body drying out). Indoor cases often display an uneven distribution of moist decomposition (i.e., putrefactive changes) and desiccation changes, making them challenging to classify, quantify, and link to a PMI.

It is known that the microorganisms present in a body (e.g., in the gastro-intestinal tract) start to migrate and invade surrounding tissues after death, as well as spreading throughout the body via the lymphatic and vascular systems. However, the tissues are believed to remain free from microorganisms for the first 24 h after death [23]. Studies have indicated that mummified tissue is free from microorganisms, meaning that the desiccation process leading to mummification takes place without putrefaction [36]. This suggests that the desiccation process begins soon after death, which is possible in artificial mummification, like that which Egyptian mummies were subjected to. Natural mummification in an indoor setting requires a rapid onset of tissue desiccation to completely inhibit microbial activity and optimize tissue preservation. In our dataset, the cases with extensive desiccation still exhibited signs of putrefaction. This indicates that the tissue had been degraded due to autolytic and putrefactive changes that occurred prior to desiccation, or in some cases were active at the same time as the process of desiccation. An experimental study of the ancient Egyptian mummification method with natron, using a donated human leg, followed the desiccation process during 208 days. Greenish discoloration of the skin and skin slippage was noted early (day 11) [36]. This is a sign of the first stage of decomposition, where sulfhemoglobin, a green pigment, is formed [37]. However, no other signs of putrefaction were seen, indicating that the natron had hampered the activity of anaerobic bacteria. The study also showed an absence of fungi and bacteria, supporting the idea that the desiccation of the leg took place without putrefactive changes [36]. During the experiment, the skin changed color to yellowish with green, brown, and red patches (day 40), and later became leathery and brownish (day 60). Desiccation seemed to start at the foot and at the end of the study (day 208) the foot, but not the thigh, was mummified [36]. In our study, it was evident that the feet (and hands) had mummified appearances and that the desiccation process had not reached the same stage in the thighs (or upper arms). We saw soft tissue desiccation mainly in feet and hands, followed by lower limbs and lower arms (Fig. 4). The experimental mummification was carried out in an enclosed indoor environment with similar temperature as in our indoor cases (i.e., in their experiment, the ambient temperature was + 19.9 ± 2.3 °C). We may assume that in our cases with evident skin and soft tissue desiccation, the process of tissue dehydration must have started soon after death due to local factors (with an effect comparable to that of natron) hampering the spread of anaerobic bacteria, thus inhibiting putrefaction. This would complicate PMI estimation, as one seldom knows to what extent putrefactive changes are repressed (i.e., the spread of anaerobic bacteria is inhibited) in a specific case. The effect on the rate of decomposition is unknown. Calculating the percentage of body surface desiccated would not give a resolution high enough to use in a model for estimation of PMI. Additional information would be needed.

### Association between the extent of desiccation and PMI

Our results indicated that cases with leathery desiccated skin generally had a longer PMI than cases with parchment-like desiccated skin, even when we included soft tissue desiccation in the comparison analysis. As shown in Fig. 5, cases with exclusively parchment-like desiccated skin more often had a PMI < 10 days, as compared with cases with exclusively leathery desiccated skin. However, the overall PMI range for leathery desiccated skin was 6–217 days and that for parchment-like skin was 3–180 days. The majority of cases displayed both leathery and parchment-like skin. A total of 21 cases exhibited 100% coverage of desiccated skin, with PMI varying between 18 and 217 days. The cases displayed heterogenic decompositional changes, and signs of moist decomposition were still evident, despite their exterior desiccation. In an article by Leccia et al. [8], it was seen that desiccation could be an early event indoors, which was supported by our findings. Our results also showed the problems of estimating PMI based on visual interpretation of desiccation degree or type. Thus, making comparisons between our findings and those in other published articles can be problematic, due to possible differences in classification of extensive or complete mummification.

#### Effect of subcutaneous fat on parchment-like desiccation

The amount of subcutaneous fat may play a role in the development of parchment-like desiccation, as we observed this type of desiccated skin mainly within the upper arms, abdomen, and thighs – anatomical areas associated with higher amounts of subcutaneous fat. Lipolysis may release liquid fat which diffuses up through the dermis and epidermis, creating the visual translucent effect of the parchment-like desiccated skin. When this skin dries out, it will look different from leathery desiccated skin. In other words, the processes behind the development of these two types of desiccated skin may differ with the anatomical region, as well as being affected by other internal or external factors.

### Effects of clothing on desiccation

In our study, we tested use of Guerra’s clothing score to quantify the presence of textiles and study if a possible insulation effect from clothing correlated with the development of desiccated skin [33]. Guerra’s scoring method may not have been the optimal choice for our dataset, since it does not take into consideration the different textiles’ capacities to absorb water from a decomposing body. Evaluating the effect of one or more layers of textiles on the body did not yield any conclusive results when analyzing the whole body and the upper body/torso. However, when comparing any coverage (partial or complete) of the legs with naked legs, the presence of textiles had a significant decelerating effect on the extent of desiccation. This may be due to anatomical differences or the inherent ability of textiles to absorb or retain moisture. Several studies evaluating the effect of clothing on the decomposition process and/or desiccation have been carried out, but usually on small data samples and often on animal models [38–43], with several possible sources of errors (such as weather, measurement of temperature, quantification of decomposition). No studies, except those carried out in a controlled laboratory environment, are in an indoor setting. The published results vary greatly and are therefore difficult to compare with ours.

### Methodological considerations

To our knowledge, this is the first observational study of desiccation of forensic importance in an indoor setting. Our dataset was comprised of authentic forensic autopsy cases with some inherent uncertainties, e.g., concerning PMI, temperature, and humidity at the sites where the bodies were found. We limited our study to visual assessment of the body exterior, using the “rule of nine” to calculate the extent of body surface affected by desiccation. Analyzing the actual water content of tissues would be interesting. This, we think, would cast further light on the desiccation process. The cases were collected nonconsecutively and some bias may exist. Desiccation seems to appear in approximately 15–20% of the forensic autopsy cases found indoors and with signs of decomposition (at our department). Measurements of temperature and humidity at the death scene might have provided more detailed information useful in the analysis of desiccation changes. Information about the location/position of the dead body at the scene of death, for example, in direct sunlight from a window or an open balcony door may also be of interest. In spite of these challenges, forensic autopsy case datasets can be valuable sources of information. Longitudinal studies (in a controlled setting) might bring more clarity, and a scoring model like that proposed by Connor et al. [9] might be achievable for indoor cases.

## Conclusions

Cases with desiccation have been thought of as outliers due to prolonged periods of supposedly hampered decomposition or have been considered too homogeneous to be used in a predictive model to estimate PMI [9]. Our study indicated a larger variation, with heterogeneous desiccated cases, at least in an indoor setting, and varied repressive effects on the decompositional process, i.e., putrefaction. Using the “rule of nines,” we assessed the skin desiccation distribution and observed a broad range of PMIs at similar percentages of desiccated skin surface. It appeared that both anatomical region and presence/absence of textile coverage had effects on the distribution and pattern of desiccation. We have probably observed differing processes leading to parchment-like or leathery desiccated skin, or a combination of the two. Our study highlights the importance of further research into the desiccation process in a forensic perspective. We think that a way forward would be systematic evaluation and creation of a standardized method for description and quantification of the desiccation changes that have taken place in a dead body, including not only the body surface, but also the tissue and internal organs. Furthermore, a combination of visual quantification methods and biochemical methods might offer a fuller picture of the desiccation process. In this paper, we have presented a first step. The study was limited to indoor conditions, reflecting the majority of cases in our day-to-day post-mortem examination activities.

## Supplementary information

Below is the link to the electronic supplementary material.

Supplementary file1 (PDF 471 KB)

Supplementary file2 (XLSX 32 KB)

## Data Availability

All data generated or analysed during this study are included in this published article and its supplementary information files.
