# Supplementary material for: Mummification in a forensic context: an observational study of taphonomic changes and the post-mortem interval in an indoor setting
Source: Int J Legal Med. 2023 Mar 21;137(4):1077–88. doi: 10.1007/s00414-023-02986-3 (PMC10247854; doi:10.1007/s00414-023-02986-3)
Supplement: Supplementary file 1 — Supplementary file1 (PDF 471 KB) [file 414_2023_2986_MOESM1_ESM.pdf]

Supplementary information

“Mummification in a forensic context: an observational study of taphonomic changes and the post-mortem interval in an indoor setting”

International Journal of Legal Medicine

Ann-Sofie Ceciliason[1], Björn Käll[2] and Håkan Sandler[1,2]

[1]Forensic Medicine, Department of Surgical Sciences; Uppsala University, Uppsala University Hospital, SE-751 85 Uppsala, Sweden

[2]Department of Forensic Medicine; The National Board of Forensic Medicine, Box 1024, SE-751 40 Uppsala, Sweden

Corresponding author: ann-sofie.ceciliason@surgsci.uu.se

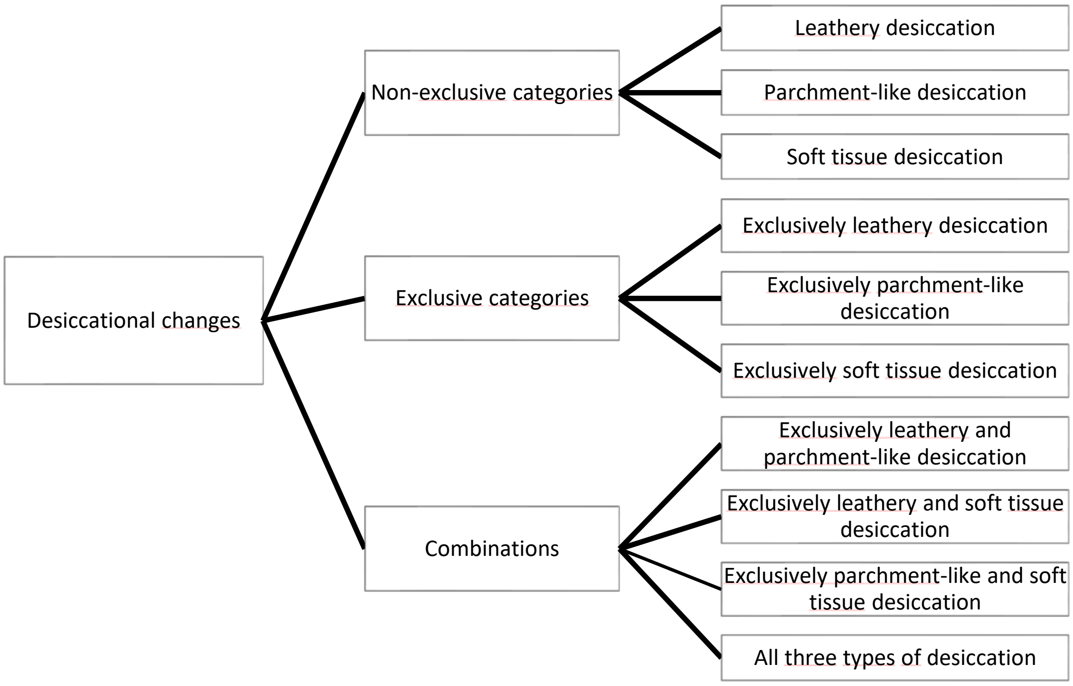

Fig. S1 Categorization of desiccation changes in the dataset

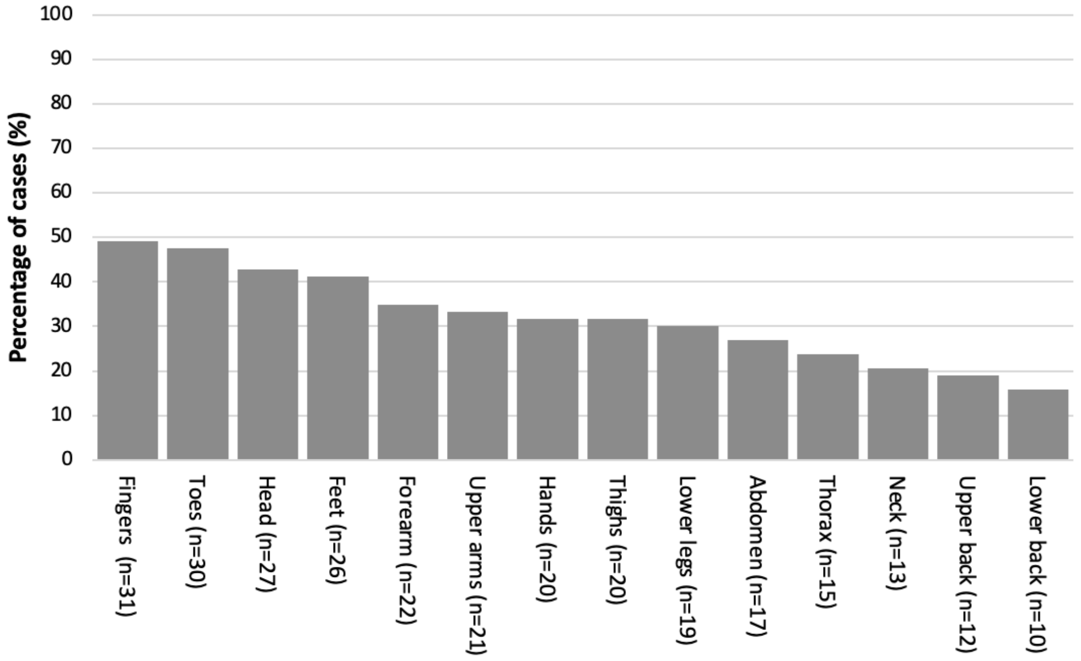

Fig. S2 Distribution of leathery desiccation of the skin across anatomical regions in 63 cases. Note that observations could be made in multiple anatomical regions for a single case

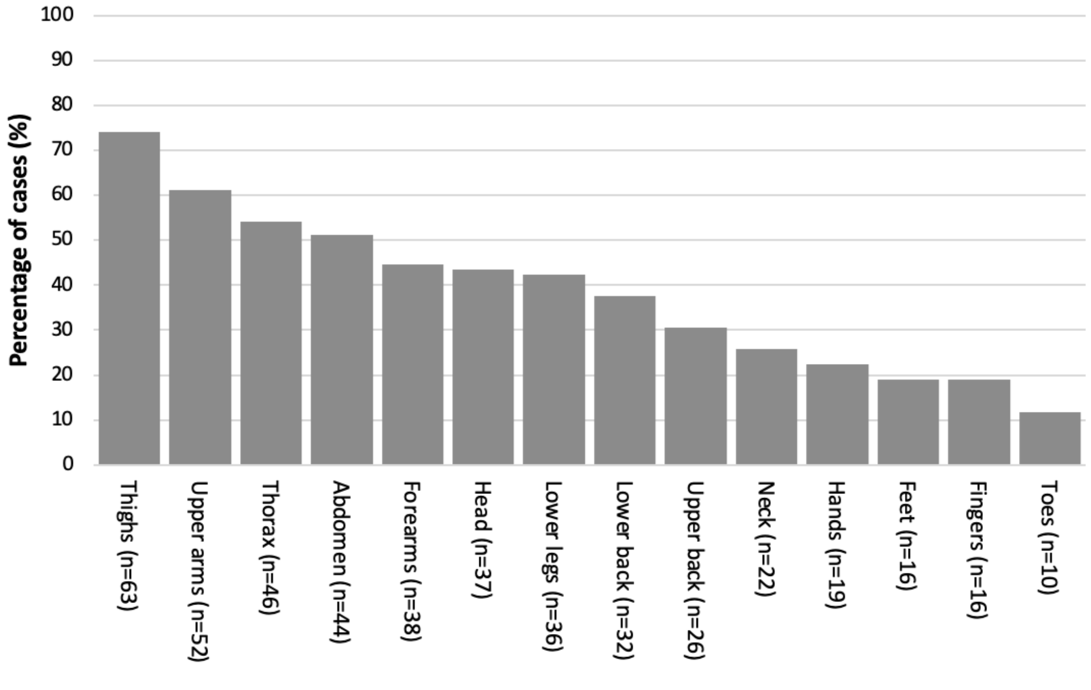

Fig. S3 Distribution of parchment-like desiccation of the skin across anatomical regions in 85 cases. Note that observations could be made in multiple anatomical regions for a single case

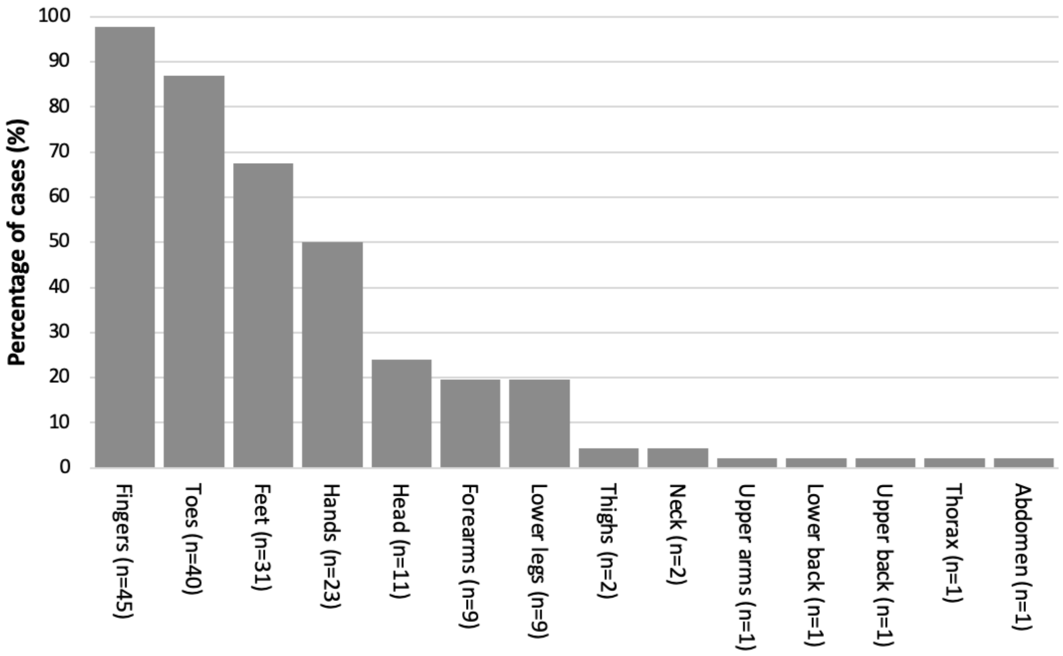

Fig. S4 Distribution of desiccation of soft tissue (i.e., subcutaneous fat and musculature) across anatomical regions in 46 cases. Note that observations could be made in multiple anatomical regions for a single case

Table S1 Guerra's original clothing score system with modifications (grey) for optimal fit to this dataset. From Guerra SC (2014) Qualifying and quantifying the rate of decomposition in the Delaware River Valley region. Publicly accessible Penn dissertations. Paper 1298. University of Pennsylvania.

**Area of the body/clothing types**	**Score**
Head	
a. Hat, Nightcap	1 point
Torso/Arms	
a. Bra	0.5 points
b. Tank top, Nightgown	0.75 points
c. Blouse, T-shirt, Shirt, Sheet	1 point
d. Robe, Long-sleeve shirt, Thermal shirt, Sweater, Sweatshirt, Pajama top, Blanket, Quilt	2 points
e. Jacket	3 points
Legs	
a. Underwear, Panties	0.5 points
b. Shorts, Boxershorts, Undershorts, Robe, Nightgown, Sheet	1 point
c. Pants, Jeans, Sweatpants, Pajama bottoms, Thermals, Blanket, Quilt	2 points
Feet	
a. Sandals, One sock	0.5 points
b. Two socks, One sneaker, Sheet	1 point
c. Two sneakers, Two boots, Two shoes, Blanket	2 points
**Our modifications**	**Score**
Whole body partially covered by a blanket	3 points
Vest	2 points
Cardigan	2 points
Towel	1 point
Jogging pants partly pulled down	1 point

Table S2 Data characteristics of cases divided into groups based on coverage of legs. Normally distributed variables described as [mean ± SD], not normally distributed variables described as [median (IQR; range)], and dichotomous variables expressed as a quotient.

Variable	***Partial or complete coverage of legs** (n = 52)*	***Complete coverage of legs** (n = 43)*	***Partial coverage of legs** (n = 9)*	***Legs naked** (n = 48)*
Female/total	11/52	8/43	3/9	11/48
Age at death (years)	62.8 ± *13.6*	62.9 ± *13.9*	*62.0 ± 12.0*	62.2 ± *12.0*
PMI (days)	21.5 *(20.0; 142.0)*	22.0 *(24.0; 140.0*)	13.0 *(14.0; 142.0)*	21.0 *(20.0; 214.0*)
Morgue time (days)	4.0 *(2.8; 17.5)*	4.0 *(3.0; 17.5)*	4.0 *(1.0; 3.0)*	5.0 (*3.0; 11.0)*
Cases with signs of insect activity	11/52	10/43	1/9	14/48
Cases with signs of mold activity	10/52	8/43	2/9	4/48

Table S3 Data characteristics of the cases in the comparative analyses, with different types of desiccation process(es) with and without (±) soft tissue desiccation process. Normally distributed variables described as [mean ± SD], not normally distributed variables described as [median (IQR; range)], and dichotomous variables expressed as a quotient.

Variable	***Exclusively leathery desiccation present*** *(n = 5)*	***Exclusively parchment-like desiccation* present** *(n = 21)*
Female/total	0/5	6/21
Age at death (years)	65.0 ± *11.2*	57.5 ± *16.7*
PMI (days)	28.0 (*35.7; 61.0*)	9.0 *(15.0; 43.0*)
Morgue time (days)	3.0 (3.5; *5.0*)	4.0 *(2.3; 11.0*)
Cases with signs of insect activity	1/5	5/21
Cases with signs of mold activity	0/5	1/21
Guerra's clothing score	2.5 *(7.0; 10.0)*	3.5 *(4.9; 10.0)*
	Leathery ± soft tissue desiccation present *(n = 17)*	***Parchment-like ±soft tissue desiccation present*** *(n = 39)*
Female/total	1/17	15/39
Age at death (years)	63.7 ± *9.9*	62.3 ± *15.0*
PMI (days)	31.0 (*29.0; 205.0*)	21.0 *(20.0; 177.0*)
Morgue time (days)	5.0 (*3.0; 5.0*)	4.0 (*2.0; 11.0*)
Cases with signs of insect activity	6/17	10/39
Cases with signs of mold activity	1/17	4/39
Guerra's clothing score	3.0 *(4.3; 10.5*)	3.4 *(5.0; 10.0*)
